# Profiling of human and microbial cell-free DNA reflects early host–pathogen interactions in sepsis

**DOI:** 10.3389/fimmu.2026.1693727

**Published:** 2026-02-10

**Authors:** Katharina Hoeter, Elmo W. I. Neuberger, Linda Marriott, Alfonso De Falco, Stephanie Wiemann, Michael K. E. Schäfer, Perikles Simon, Maïwenn Kersaudy-Kerhoas, Marc Bodenstein

**Affiliations:** 1 Department of Anesthesiology, University Medical Centre of the Johannes Gutenberg-University, Mainz, Germany; 2 Department of Sports Medicine, Disease Prevention and Rehabilitation, Johannes Gutenberg-University Mainz, Mainz, Germany; 3 Institute of Biological Chemistry, Biophysics and Bioengineering, Heriot-Watt University, Edinburgh, United Kingdom

**Keywords:** DAMPs, inflammation pathways, NEtosis, PAMPS, sepsis

## Abstract

**Objectives:**

In sepsis, circulating cell-free DNA (cfDNA) originates from host cells (damage-associated molecular patterns, DAMPs) and pathogens (pathogen-associated molecular patterns, PAMPs), contributing to immune activation and offering potential as both a biomarker and a therapeutic target. While DAMPs are thought to predominate in early sepsis, this study aimed to quantify their relative abundance and assess their correlation with inflammatory markers compared to PAMPs.

**Methods:**

In this prospective observational study, blood samples of 18 ICU patients were collected within 24 hours of sepsis diagnosis. Plasma cfDNA was analyzed via qPCR (targeting human LINE-1) and iSEP-SEQ nanopore sequencing. Human and microbial cfDNA were quantified, and method correlation was assessed using Kendall’s tau-b correlation. Associations with inflammatory biomarkers were tested using Spearman correlation analysis and group comparisons between human and non-human reads were analyzed with Pearson correlation analysis. The study received ethical approval from the Landesärztekammer Rheinland-Pfalz (Approval Number: 2020-15535).

**Results:**

cfDNA was predominantly of human origin, comprising 99.86% of classified reads, with microbial cfDNA accounting for only 0.077% (p < 0.001). qPCR-based cfDNA concentrations strongly correlated with human read counts from sequencing (τ = 0.712; p < 0.001). Human cfDNA levels were significantly associated with LDH, WBC, and CRP. Microbial cfDNA, although low in abundance, correlated with WBC, CRP, and D-dimer.

**Conclusions:**

In early sepsis, human cfDNA is markedly more abundant than microbial cfDNA. However, both exhibit strong correlations with inflammatory and tissue injury markers. These findings support a model of PAMP-triggered and DAMP-driven inflammation and identify human cfDNA as a promising biomarker and potential therapeutic target.

**Clinical Trial Registration:**

https://drks.de/search/de/trial/DRKS00025222/details, identifier DRKS-ID: 00025222.

## 1 Introduction

Sepsis is a leading cause of global morbidity and mortality, defined as a life-threatening organ dysfunction caused by a dysregulated host response to infection (1–3). A hallmark of sepsis is an exaggerated inflammatory response that, while initially aimed at controlling infection, often spirals into widespread tissue damage and organ failure (4). Despite advances in intensive care, early diagnostic tools and targeted therapies remain limited. A better understanding of the molecular mechanisms driving immune dysregulation is essential for improving patient outcomes.

**Figure 1:**
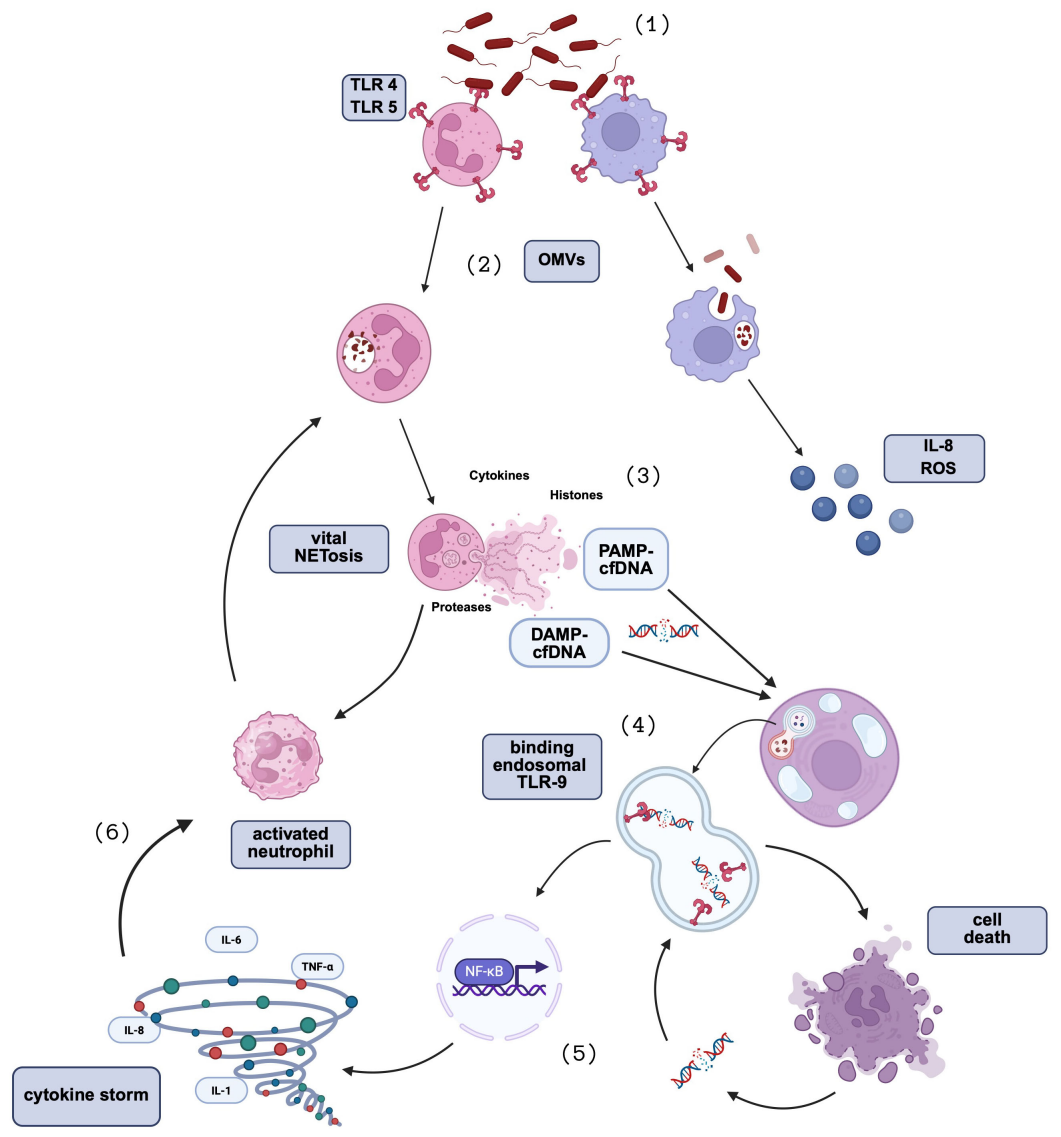
Schematic representation of the roles of PAMPs, DAMPs, and NETosis in sepsis pathophysiology. Created in BioRender. Schaefer, M. (2026) https://BioRender.com/yuwvkok. (1) During sepsis, microbial components known as pathogen-associated molecular patterns (PAMPs), including bacterial DNA, LPS, and peptidoglycans, are recognized mainly by Toll-like receptors (TLR), particularly by TLR 4 and 5, which are host pattern recognition receptors (PRRs)This interaction initiates a potent innate immune response. (2) Following PRR-mediated recognition, microbial components, including microbial DNA, are internalized by immune cells such as neutrophils and monocytes, via outer membrane vesicles (OMVs). (3) Neutrophils, activated by these PAMPs, undergo a specialized form of cell death known as NETosis, releasing neutrophil extracellular traps (NETs), which are composed of bacterial fragments (PAMPs), histones, proteases, cytokines, and cell-free DNA (cfDNA), originating from the host cells and functioning as a damage-associated molecular pattern (DAMP). (4) Both, microbial cfDNA contained within NETs (functioning as PAMPS) and predominantly host-derived cfDNA released during NETosis (functioning as DAMPS) are recognized by endosomal TLR9. While microbial cfDNA primarily contributes to initial immune activation, host-derived cfDNA promotes cellular injury, induction of apoptosis, and the release of additional DAMPs, thereby amplifying the inflammatory cascade. (5) cfDNA also triggers NF-κB activation and subsequent cytokine release, (6) which in turn further activate neutrophils and NETosis. While NETs contribute to the trapping and neutralization of pathogens, excessive NET formation contributes to endothelial damage, thrombosis, and organ dysfunction. The figure illustrates the interconnected roles of PAMPs, DAMPs, and NETosis in driving the dysregulated immune response characteristic of sepsis.

Circulating cell-free DNA (cfDNA), released during host cell injury or microbial invasion, has emerged as a potential biomarker and active mediator in sepsis (**Figure 1**). cfDNA fragments function as immunostimulatory molecules - either damage-associated molecular patterns (DAMPs) from host cells or pathogen-associated molecular patterns (PAMPs) from infectious agents – activating innate pattern recognition receptors (PRRs) such as Toll-like receptors (TLRs) (4, 5).

Although both DAMPs and PAMPs contribute to inflammatory cascade in sepsis, their relative impact remains poorly understood (6). Recent studies suggest that host-derived cfDNA may dominate, particularly in bacterial infections (7). While PAMPs initiate immune responses, sustained inflammation may be driven largely by DAMPs, including those released during neutrophil NETosis (4). However, quantitative data on DAMP vs. PAMP contributions in early sepsis is limited. In addition to triggering inflammation, cfDNA and related molecular patterns promote a procoagulant state and contribute to impaired fibrinolysis, which are central features of sepsis-associated coagulopathy and microvascular thrombosis (8).

In this study, we employed the iSEP-SEQ Microbial cfDNA Assay (9), a nanopore-based sequencing platform capable of detecting and quantifying both human and microbial cfDNA in plasma. This approach enabled high-resolution profiling of cfDNA during the early phase of sepsis (within the first 24 hours of diagnosis)—a critical window marked by immune activation and clinical decline. By correlating cfDNA composition with inflammatory markers and clinical data, we aimed to clarify the roles of host and microbial cfDNA in sepsis pathogenesis. Our findings provide new insights into cfDNA dynamics and their relevance for sepsis diagnosis and targeted treatment strategies.

## 2 Materials and methods

### 2.1 Study design and patient population

This is an exploratory, prospective observational study, with the objective to quantify the amount of human and pathogen-derived cfDNA in patients with sepsis.

The study was carried out at the Intensive Care Unit (ICU) of the Department of Anesthesiology, University Medical Center Mainz, Germany. Eligible patients were adults aged ≥18 years, recruited upon clinical sepsis diagnosis as defined by the Sepsis-3 criteria (1). Patients with conditions known to be associated with elevated baseline levels of cfDNA, such as pregnancy, breastfeeding, or active malignancy, were excluded. All laboratory parameters, including IL-6, were obtained from routine clinical measurements and extracted from the hospital electronic medical records; no additional laboratory assays were performed specifically for this study. To minimize potential confounding by prior antimicrobial exposure, blood samples were collected at the earliest feasible timepoint following clinical suspicion of sepsis or immediately after ICU admission for the septic episode. Patients were observed for a period of 30 days following enrollment.

### 2.2 Sample processing and microbiological analysis

EDTA (ethylenediaminetetraacetic acid) blood samples were collected from each patient and processed within three hours of collection. Samples were first centrifuged at 3,800 × g for a period of 10 minutes, followed by a second centrifugation step at 12,000 × g for a duration of 10 minutes at a temperature of 20°C. The plasma obtained was then stored at -80°C until further analysis.

Subsequently, two complementary analyses of cfDNA were performed: quantitative polymerase chain reaction (qPCR) and a microfluidic-based sequencing approach. qPCR was conducted at the Department of Sports Medicine, Disease Prevention and Rehabilitation, Johannes Gutenberg University Mainz, Mainz, Germany. This analysis was based on the protocol established by Neuberger et al. (10), targeting 90 bp and 222 bp amplicons of human long interspersed nuclear elements (LINEs) from the L1PA2 family (11).

For the microfluidic-based sequencing analysis, plasma samples were shipped on dry ice to the Institute of Biological Chemistry, Biophysics and Bioengineering, Heriot-Watt University, Edinburgh Campus, United Kingdom. After thawing, cfDNA was extracted using a proprietary microfluidic technique described in (9). Briefly, cfDNA was extracted from 1 mL of plasma on a custom cassette and automation platform. Extracted eluates were stored at -20°C until library preparation. A negative extraction control (DPBS buffer processed in parallel with the plasma samples) was included to assess background contamination; no positive spike-in controls were performed as part of this study. Sequencing libraries were prepared using the genomic DNA ligation kit (SQK-LSK114, Oxford Nanopore Technologies) using a method based on the Flongle Ligation sequencing V14 protocol (Oxford Nanopore Technologies). SPRIselect beads (Beckman Coulter) were used in place of the kit beads at ratios optimized for the retention of short DNA fragments. DNA input for library preparation was sample dependent and varied between 18ng and 100ng. Although we aimed for between 50-100ng of DNA in the starting sample, variations in the DNA concentration of the eluate and the eluate volume obtained from the extraction did not always allow for this. Libraries were sequenced on a MinION Mk1C (MinKNOW software v23.07.12 or v24.11.8, Oxford Nanopore Technologies) using a Flongle flow cell (FLO-FLG114, Oxford Nanopore Technologies) for two hours. All samples were sequenced using identical instrument settings and run times; variation in organism-specific sequencing depth reflects biological differences in cfDNA abundance rather than technical variability. For samples sequenced using MinKNOW software v23.07.12, fast5 files were re-basecalled using MinKNOW software v24.11.8 (Dorado v7.6.7, High Accuracy Basecalling) before analysis. Sequence analysis was performed using the EPI2ME metagenomics workflow (v2.12.1, ONT) utilizing Kraken2 software for the taxonomic classification of sequencing reads (Wood, D.E., Lu, J. & Langmead, B. Improved metagenomic analysis with Kraken 2. Genome Biol 20, 257 (2019) (12).

### 2.3 Data analysis

Statistical analysis was performed using IBM SPSS Statistics (Version 29.0.0.0, IBM Corp., Armonk, NY). Continuous variables were reported as mean ± standard deviation (SD) if normally distributed, or as median with interquartile range (IQR) if not normally distributed; categorical variables as frequencies and percentages.

Kendall’s tau-b correlation was used to assess the relationship between log-transformed cfDNA values (qPCR vs. sequencing). Spearman’s rank correlation was used for all associations involving cfDNA concentrations, cfDNA integrity indices, and inflammatory biomarkers, as these variables were not normally distributed. Pearson correlation was applied exclusively to assess associations between log-transformed human and non-human sequencing reads. Paired t-tests assessed differences between human and non-human reads.

### 2.4 Ethical issues

The study was approved by the Landesärztekammer Rheinland-Pfalz (Number: 2020-15535), registered in the German Clinical Trials Register (DRKS-ID: 00025222; Amendment), and conducted under informed consent under the Declarations of Helsinki.

## 3 Results

**Figure 2:**
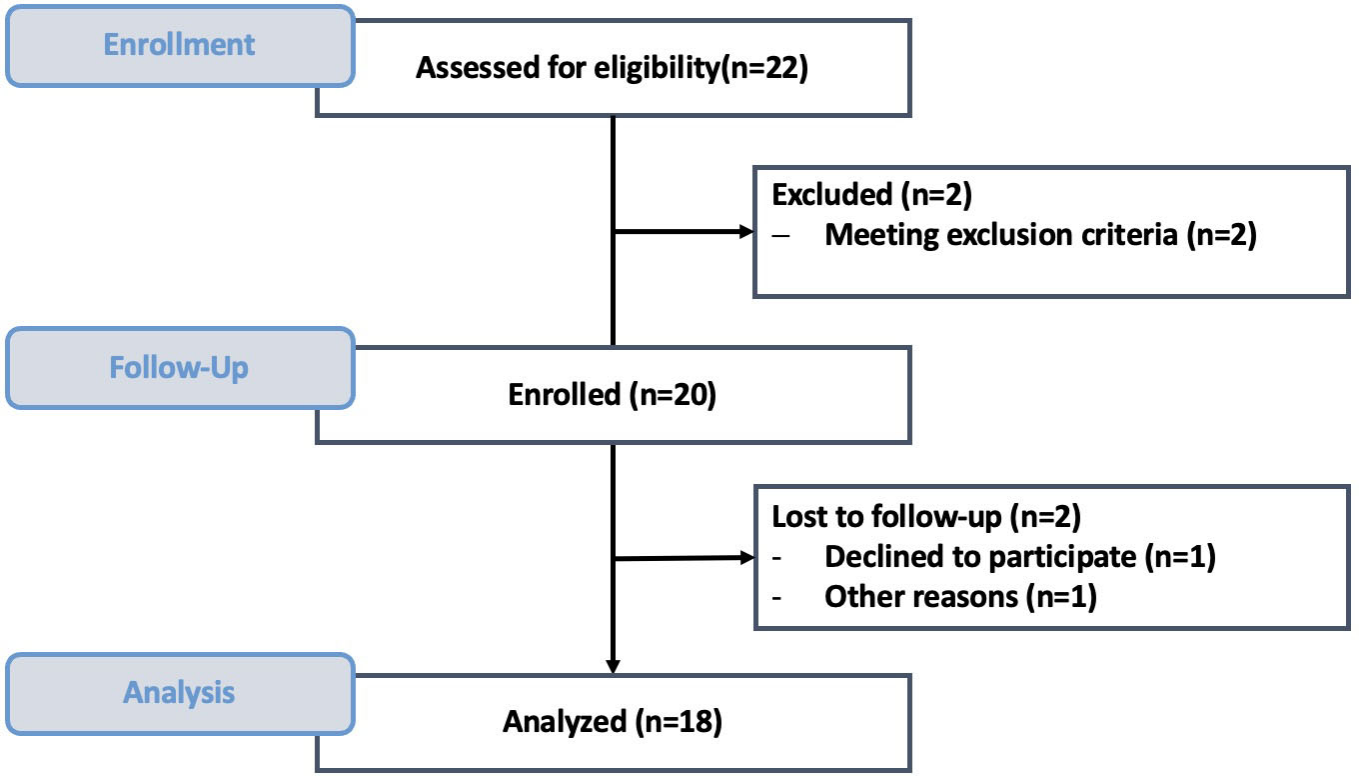
Flow diagram of patient recruitment and inclusion.

Between October 2023 and July 2024, 54 cfDNA analyses were performed in 18 patients diagnosed with sepsis (median age 74.5 years; 50% female). Baseline data showed marked inflammation and organ dysfunction, with a mean SOFA score of 8.53 ± 5. **Figure 2** illustrates patient inclusion; **Table 1** summarizes clinical and demographic characteristics. A detailed overview of the sepsis focus, clinical specimen types, and corresponding microbiological findings for all patients is provided in **Table 2**.

**Table 1:** Demographic and clinical baseline characteristics of patients with sepsis diagnosis.

Parameter	Sepsis cohort
Age, years, median (± SD)	74.5 (13.43)
Sex, n (%)	
- Female	9 (50)
- Male	9 (50)
BMI, kg/m^2^, mean (±SD)	26.1 (6.1)
Pre-existing comorbidities	n (%)
Cardiovascular disease	10 (55.6)
Arterial hypertension	12 (66.7)
Chronic kidney disease	5 (27.8)
Chronic pulmonary disease	9 (50.0)
Immunologic disease	4 (22.2)
Diabetes mellitus type II	9 (50.0)
Obesity	5 (27.8)
Laboratory parameters at enrollment	mean (±SD)
CRP [>5 mg/L]	210 (106.6)
LDH [>245 U/L]	359 (309.3)
PCT [>0.5 ng/mL]	6.98 (10.8)
Creatinine [>1.18 mg/dL]	1.89 (1.2)
Urea [>26 mg/dL]	44 (32.3)
eGFR < 62 ml/min/L.73m^2^	48 (35.0)
Bilirubin [>1.2 mg/dL]	1.2 (1.7)
D-Dimer [>0.5 mg/L]	9.44 (11.6)
Interleukin-6	180 (170.3)
CK [>200 U/L]	306 (418.8)
Troponin [>24 pg/mL]	630.6 (1142.9)
WBC [>10/nL]	15.4 (9.3)
Platelets [<165/nL]	203 (136.9)
Lactate [>1.6 mmol/L]	2.4 (3.3)
Hemoglobin [<11.7 g/dL]	9.3 (1.3)
pH	7.36 (0.1)
pCO_2_ [>48 mmHg]	40 (12.6)
pO_2_ [<83 mmHg]	99 (36.6)
sO_2_ [<95 %]	96.3 (2.4)
HCO_3_ [<22.5 mmol/L]	22.1 (4.4)
BE [<-1.5 mmol/L]	-2.9 (5.4)
BNP [>125 pg/mL]	5124 (6987.8)
Clinical outcome parameters	mean (± SD)
Ventilation days	9 (15.9)
ICU LOS, d	15.61 (20.3)
Hospital LOS, d	26.56 (19.3)
GCS	10.67 (4.9)
Temperature, °C	36.92 (0.8)
SOFA	8.53 (5.1)
30-day mortality, n (± SD)	7 (39%)

BE, base excess; BMI, Body Mass Index; BNP, B-type natriuretic peptide; °C, degree Celsius; CK, creatinine kinase; CRP, C-reactive Protein; d, days; eGFR, estimated glomerular filtration rate; GCS, Glasgow Coma Scale; HCO_3_, bicarbonate; L, liter; LDH, Lactate dehydrogenase; LOS, length of stay; mg, milligram; mL, millilitre; mmHg, milliliters of mercury; mmol, millimole; nL, nanoliter; pg, pictogram; pCO_2_, partial pressure of carbon dioxide; PCT, Procalcitonin; pH, power of hydrogen; pO_2_, partial pressure of oxygen; SD, standard deviation; sO_2_, saturation of oxygen; SOFA-Score, Sequential Organ Failure Assessment Score; U, Units; WBC, white blood cell count; y, years.

**Table 2:** Overview of sepsis focus, clinical specimen types, and microbiological findings.

ID	Sepsis focus	Clinical specimen type	Microbiological testing results
1	Sepsis due to a recurrence of Clostridium difficile colitis	Stool Culture	Clostridium difficile
		Native Aspirate	Streptococcus anginosus Bacteroides fragilis Corynebacterium striatum
		Bronchial Secretion	Acinetobacter junii
		Tracheal Secretion	Candida species
		Rectal Swab	Enterococcus faecium
		Ascites Fluid	Enterococcus faecium Candida species
2	Hospital-aquired pneumonia	Urine Culture	Pseudomonas aeruginosa
4	Hospital-aquired pneumonia	Pacemaker Lead (part 2)	Cutibacterium acnes
5	ARDS (aspiration)	CVC Culture (aerobic/anaerobic)	Clostridium symbiosum
		CVC Culture (aerobic/anaerobic)	Clostridium symbiosum Eggerthella lenta
		Artery Culture (aerobic/anaerobic)	Staphylococcus capitis
		Artery Culture (aerobic/anaerobic)	Clostridium symbiosum
		Blood Culture (aerobic/anaerobic)	Escherichia colli Clostridium symbiosum Solobacterium moorei
		Intraoperative Abdominal Swab (Douglas)	Escherichia colli Klebsiella pneumoniae complex Streptococcus anginosus Streptococcus militis Bacteroides ovatus Bacteroides thetaiotaomicron
		Tracheal Secretion	Candida species Aspergillus fumigatus
		PiCCO Line Culture (aerobic/anaerobic)	Clostridium symbiosum
7	Urosepsis	Urine Culture (catheter)	Escherichia colli
		CVC Culture (aerobic/anaerobic)	Staphylococcus hominis Staphylococcus epidermidis
8	Urosepsis	Drainage Fluid	Klebsiella oxytoca
		Drainage Fluid	Enterococcus faecium
9	Hospital-aquired pneumonia	Induced Sputum	Escherichia colli Candida species
10	Mitral valve endocarditis	Tracheal Secretion	Candida species Klebsiella oxytoca Serratia marcescens
11	Hospital-aquired pneumonia	Artery Culture (aerobic/anaerobic)	Staphylococcus haemolyticus
		Tracheal Secretion	Citobacter coseri Candida species
		Rectal Swab	Enterococcus faecium
		Multiplex PCR Pneumonia Panel	Escherichia colli Coronavirus RNA
12	Lower limb gangrene	Wound Swab (lower limb)	Bacteroides fragilis Morganella morganii Proteus vulgaris Stenotrophomonas maltophila
		Wound Swab (lower limb)	Enterococcus faecium Corynebacterium jeikeium
14	Gangrene associated with diabetic foot syndrome	Blood Culture (aerobic/anaerobic)	Bacteroides fragilis
		Tracheal Secretion	Candida species
		Wound Swab (lower limb)	Escherichia colli Pseudomonas aeruginosa Enterococcus avium Coagulase-negative Staphylococcus Bacteroides fragilis Actionmyces turicensis Bacteroides ovatus
15	Community-aquired pneumonia with immobilization trauma	Urine Culture (catheter)	Klebsiella oxytoca Escherichia colli
		Wound Swab (lower limb)	Staphylococcus aureus Corynebacterium species Enterobacteriaceae
18	HAP (aspiration)	Pleural/Empyema Fluid Culture (aerobic/anaerobic)	ESBL Klebsiella pneumoniae complex
		Wound Swab (decubitus)	Escherichia colli Pseudomonas aeruginosa Bacteroides fragilis Morganella morganii
19	Four-quadrant peritonitis	Intraoperative Abdominal Swab	Escherichia colli Citrobacter freundii complex Bacteroides xylanisolvens Flavonifractor plautii
		Tracheal Secretion	Escherichia colli Candida species
20	Abdominal focus	Urine Culture	Escherichia colli
		CVC Culture (aerobic/anaerobic)	Escherichia colli
		Artery Culture (aerobic/anaerobic)	Escherichia colli
		Intraoperative Abdominal Swab	Escherichia colli Cutibacterium avidum Staphylococcus epidermidis Enterococcus faecalis
		Abdominal Fluid (urinary bladder)	Escherichia colli Enterococcus faecalis Actinobaculum schaalii
21	Urosepsis	CVC Culture (aerobic/anaerobic)	Klebsiella pneumoniae complex
		Native Renal Aspirate	Klebsiella pneumoniae complex
		PiCCO Line Culture (aerobic/anaerobic)	Klebsiella pneumoniae complex
22	Abdominal infectious focus	Shaldon Catheter Culture	Enterococcus faecium
		Intraoperative Abdominal Swab	Enterococcus faecium Candida albicans

ARDS, Acute Respiratory Distress Syndrome; CVC, Central venous catheter; ESBL, Extended-spectrum β-lactamase; HAP, Hospital-acquired pneumonia; PCR, Polymerase chain reaction.

**Figure 3:**
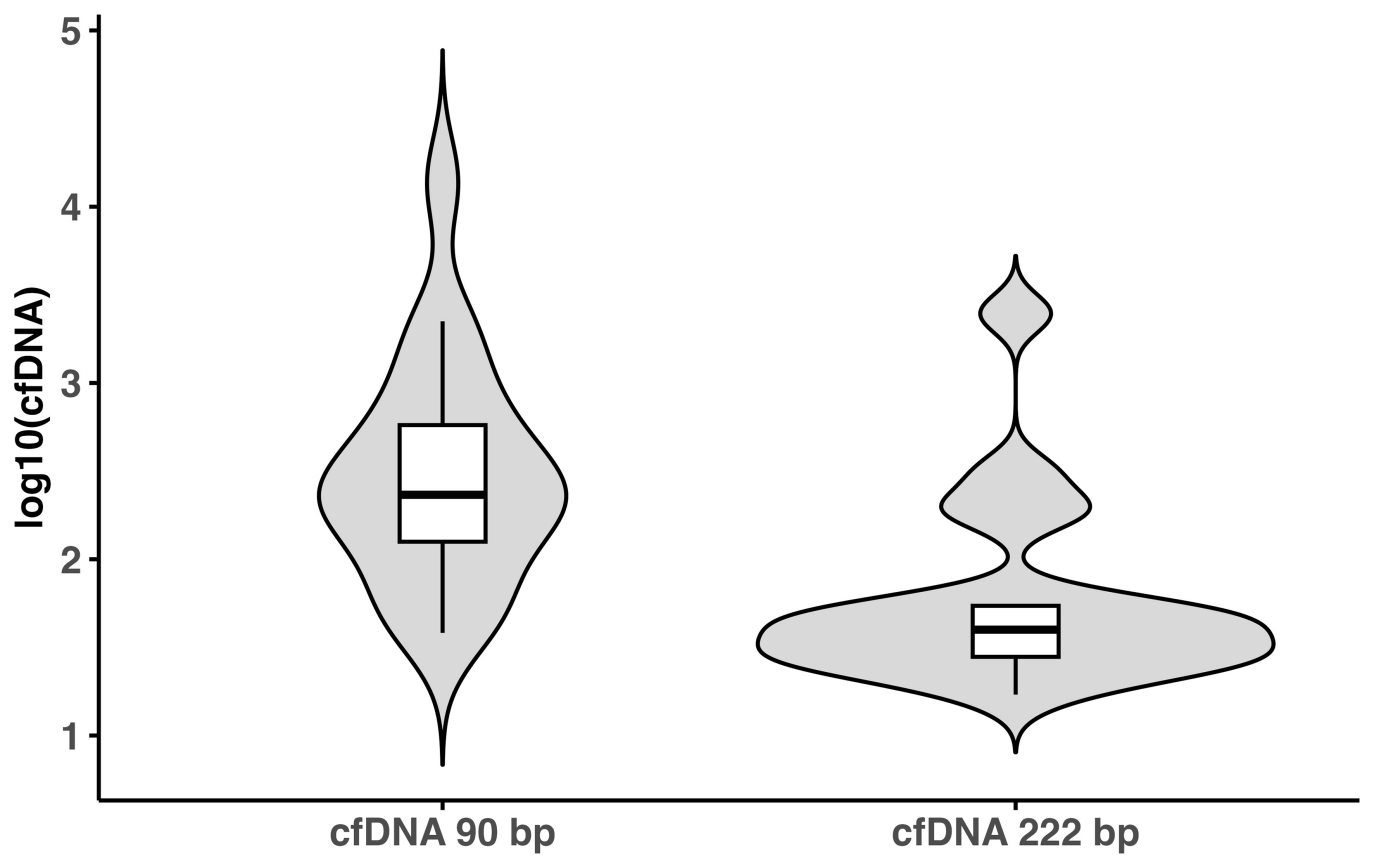
Violin plots illustrating the distribution of cfDNA concentrations for the 90 bp and 222 bp amplicons (log10-transformed). Boxplots are embedded within each violin to show median and interquartile range.

qPCR revealed median cfDNA concentrations of 232.298 ng/mL (90 bp) and 39.857 ng/mL (222 bp), with an integrity index of 0.195. The iSEP-SEQ assay yielded a median of 180187.5 total reads per sample, 99.86% of which were human and 0.077% non-human. **Table 3** summarizes cfDNA fragment types and sequencing read distributions and **Figure 3** provides a visual representation of cfDNA concentration variability using violin plots.

**Table 3:** Overview of cfDNA types and read distribution.

cfDNA classification	cfDNA-concentration, ng/mL (Median [IQR])
qPCR	
cfDNA 90 bp, ng/mL	232.298 (547.67)
cfDNA 222 bp ng/mL	39.857 (58.52)
Integrity Index	0.195 (0.22)
iSEP-SEQ Microbial cfDNA Assay	cfDNA-number of reads (Median [IQR])
Total reads	180187.5 (140279.25)
Unclassified reads	55078.5 (30873.75)
Classified reads	121168 (122494)
Human reads	121079.5 (122437.75)
Non-human reads	89.5 (73.25)
	Mean (± SD)
Percentage human reads	99.92 (0.022)
Percentage of non-human reads	0.077 (0.022)

bp, base pairs; cfDNA, cell-free DNA; mL, millilitre; ng, nanogram; qPCR, quantitative polymerase chain reaction; SD, standard deviation.

### 3.1 qPCR and iSEP-SEQ method correlation

**Figure 4:**
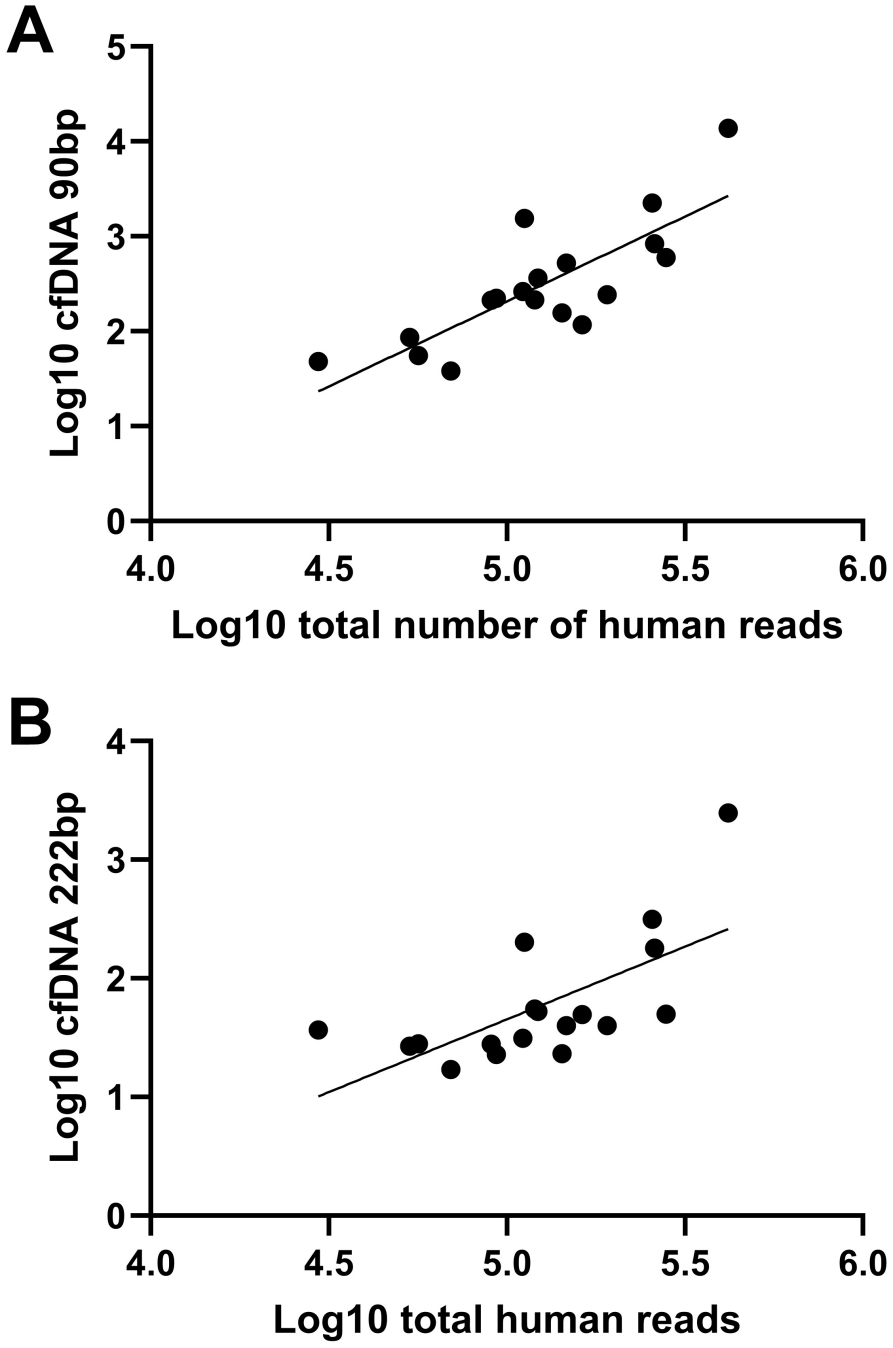
Scatter plot of log-transformed cfDNA concentration [**(A)** 90 bp and **(B)**] 222 bp) versus log-transformed total human read count. Each point represents one sample.

**Figure 4** shows a scatterplot illustrating the correlation between cfDNA quantification by qPCR and the iSEP-SEQ assay. Kendall’s tau-b correlation was 0.712 (p < 0.001) for the 90 bp fragments and 0.634 (p < 0.001) for the 222 bp fragments, indicating statistically significant and consistent agreement between the two measurement methods across both fragment sizes.

### 3.2 cfDNA read composition

**Figure 5:**
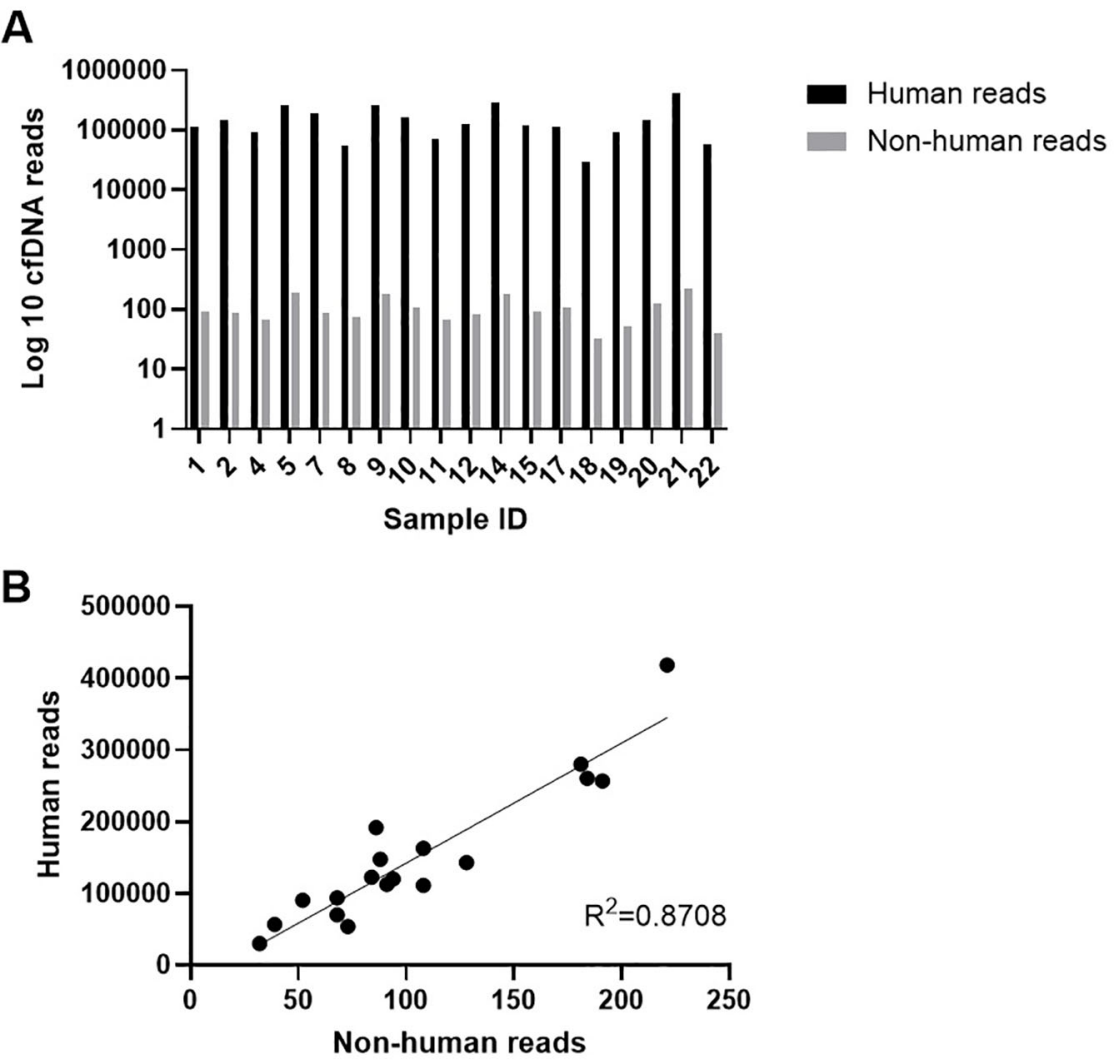
**(A)** Grouped bar chart showing the log-transformed number of reads assigned to human and non-human cell-free DNA (cfDNA) across samples. Patient IDs range from 1 to 22; however, IDs 3, 6, 13, and 16 were excluded during screening (see patient flow diagram) and therefore do not appear in the figure. **(B)** Scatter plot displaying the correlation between the number of non-human reads(x-axis) and human reads (y-axis) across individual samples, with corresponding R² value. Each dot represents a single sample.

A grouped bar chart (**Figure 5A**) visualized log-transformed human and non-human cfDNA reads. An independent t-test showed a significant difference in abundance (p < 0.001). Pearson correlation analysis (**Figure 5B**) showed a strong positive association between human and non-human reads (r = 0.87, p < 0.001).

### 3.3 cfDNA and inflammatory markers

Correlations between cfDNA metrics and laboratory parameters were assessed using Spearman’s coefficient (**Table 4**). cfDNA 90 bp levels correlated significantly with CRP (p = 0.005) and WBC (p = 0.014); 222 bp fragments also correlated with WBC (p = 0.003). The cfDNA integrity index showed a significant negative correlation with CRP (p = 0.039), while no associations were observed with WBC, IL-6, PCT, or D-dimer. Total human reads were associated with WBC (p = 0.03) and LDH (p = 0.012). Non-human reads showed significant correlations with CRP (p = 0.013), WBC (p = 0.003), and D-dimer (p = 0.017). Associations with other markers such as PCT and IL-6 were weaker and not statistically significant. No relevant correlations were found with lactate or platelet counts for any cfDNA measure, indicating selectivity of cfDNA associations with inflammatory and injury markers.

**Table 4:** Correlation between 90 bp and 222 bp cfDNA, human and non-human reads, and inflammatory markers at the time of sample collection (Spearman's rho).

	cfDNA 90bp		cfDNA 222bp		Integrity index		Human reads		Non-human reads	
Laboratory parameters	Cor. coeff.	p-value	Cor. coeff.	p-value	Cor. coeff.	p-value	Cor. coeff.	p-value	Cor. coeff.	p-value
CRP	0.63	0.005*	0.29	0.24	-0.49	0.039*	0.38	0.12	0.58	0.013*
PCT	0.42	0.09	0.22	0.39	-0.22	0.37	0.38	0.12	0.44	0.07
LDH	0.28	0.26	0.32	0.2	0.08	0.75	0.58	0.012*	0.44	0.07
WBC	0.57	0.01*	0.63	0.005*	-0.19	0.44	0.51	0.03*	0.65	0.003*
IL-6	0.49	0.07	0.44	0.12	-0.26	0.36	0.24	0.41	0.24	0.42
D-Dimer	0.38	0.14	0.43	0.1	0.04	0.87	0.48	0.06	0.58	0.017*
Lactate	0.4	0.1	0.35	0.15	-0.15	0.54	0.09	0.7	0.03	0.91
Platelets	-0.15	0.56	0.05	0.86	0.14	0.59	-0.05	0.84	0.06	0.82

bp, base pairs; Cor. Coeff., correlation coefficient; CRP, C-reactive Protein; IL-6, Interleukin-6; LDH, Lactate dehydrogenase; PCT, Procalcitonin; WBC, white blood cell count.

## 4 Discussion

In this study cfDNA was quantified in patients in the early stages of sepsis by employing both qPCR and the iSEP-SEQ Microbial cfDNA sequencing assay. The findings demonstrated a strong correlation and close agreement between the two methods of determining human cfDNA values. The iSEP-SEQ Microbial cfDNA sequencing assay further revealed that the majority of cfDNA reads were of human origin (mean 99.86% ± 0.022), with non-human microbial reads representing only a small fraction (0.077% ± 0.022). However, both cfDNA fragment concentrations and sequencing-based read distributions demonstrated distinct associations with inflammatory and cell injury markers.

These results show the quantitative dominance of human cfDNA in comparison to bacterial cfDNA during the critical early 24 hours of sepsis, thus suggesting their involvement in inflammatory dysregulation. These findings emphasize the potential of human cfDNA as a clinically relevant biomarker and underscore the importance of targeting host-derived inflammation in future therapeutic strategies.

### 4.1 Concordance of qPCR and iSEP-SEQ

The strong linear correlation observed between cfDNA quantification by qPCR and two-hour iSEP-SEQ Microbial cfDNA sequencing assay (9) indicates that both methods provide comparable estimates of total human cfDNA concentration in septic patients. This is in line with previous studies showing that while qPCR offers high sensitivity and speed for cfDNA detection (10), sequencing-based approaches provide broader information, including fragment diversity and microbial taxonomy (11, 13).

Unlike qPCR, the iSEP-SEQ assay enables the simultaneous detection of microbial cfDNA from bacteria, viruses, and fungi — an advantage in diagnosing infections and understanding host–pathogen dynamics. Taken together, these findings support the use of iSEP-SEQ not only as a research tool but also as a clinically relevant method for cfDNA profiling in sepsis. Its agreement with qPCR and added capacity for microbial detection position it as a promising approach for both biomarker development and pathogen identification in critically ill patients (14, 15).

### 4.2 Early Sepsis: PAMP trigger, DAMP dominance

The cfDNA profiles analyzed in this study reflect the immune landscape within 24 hours of sepsis diagnosis, i.e., before significant therapeutic intervention and prior to full escalation of the inflammatory response. In line with a previous study (7) we observed a clear predominance of host-derived over microbial cfDNA in all patient samples, with a statistically significant difference in read abundance. Despite this imbalance, the strong positive correlation between the two cfDNA types suggests a biologically coordinated release pattern.

This supports a model in which PAMPs initiate immune activation via pattern recognition receptors (PRRs), triggering downstream signaling cascades that result in host tissue damage and subsequent DAMP release, including nuclear and mitochondrial cfDNA (16).

The data suggests that DAMP release scales with the intensity of the PAMP-triggered immune response. This is consistent with previous findings showing that microbial stimuli (e.g., through LPS or bacterial DNA) induce secondary tissue injury and cfDNA release. These DAMPs further amplify inflammation through NF-κB and TLR9 signaling pathways (16, 17). Based on our findings, it is conceivable that even very small amounts of microbial cfDNA act as an initial trigger, while the magnitude of the subsequent host-derived cfDNA release reflects the strength of the amplified immune response. The observed correlation between microbial and host cfDNA may therefore represent a hypothesis-generating indication of this two-step mechanism, where pathogen-derived signals initiate immune responses and host-derived cfDNA contributes to their escalation.

Taken together, these findings underline the central role of DAMP-driven amplification in early sepsis. While microbial cfDNA is detectable and likely essential for initiating immune recognition, the disproportionate abundance of host-derived cfDNA highlights its potential as a therapeutic target for limiting hyperinflammation and preventing organ dysfunction. However, the precise quantitative relationship between microbial cfDNA as an initiating trigger and host cfDNA as an amplified DAMP response remains to be determined in future studies.

### 4.3 cfDNA Dynamics and implications for host–pathogen interactions

The importance of cfDNA dynamics for understanding host–pathogen interactions in sepsis is supported by longitudinal observations demonstrating that cfDNA levels and fragmentation patterns evolve substantially during critical illness. In the COVSEP-study, cfDNA concentrations measured early after ICU admission already reflected acute immune activation, but longitudinal profiling revealed that cfDNA trajectories diverged over time, with persistently elevated cfDNA levels observed in patients with ongoing immune dysregulation. In parallel, changes in cfDNA fragmentation patterns suggested shifts in dominant cell death mechanisms, consistent with transitions between apoptosis, NETosis, and necrotic tissue injury (18).

These findings have direct implications for host–pathogen interactions. Early cfDNA release likely reflects PAMP-triggered immune activation, whereas sustained host-derived cfDNA over time indicates continued DAMP-driven amplification of inflammation despite pathogen control. Such dynamics suggest that cfDNA not only mirrors pathogen burden but also captures the balance between effective immune resolution and self-perpetuating host-mediated tissue damage.

In the context of the present study, which focuses on the first 24 hours of sepsis, the observed associations between cfDNA measures and inflammatory and tissue injury markers indicate that DAMP-driven processes are already engaged very early after disease onset. Based on prior longitudinal observations, it is plausible that subsequent cfDNA trajectories would distinguish patients in whom early host–pathogen interactions resolve from those who progress toward sustained immune dysregulation and organ dysfunction. Integrating early cfDNA composition with longitudinal cfDNA dynamics may therefore provide a more comprehensive mechanistic view of sepsis progression.

The present study cohort predominantly consisted of patients with bacterial sepsis, and cfDNA analyses were not stratified by pathogen type. Consequently, pathogen-specific differences in cfDNA release, fragmentation patterns, and immune activation could not be systematically assessed. This is particularly relevant for viral infections, which may exhibit distinct cfDNA dynamics compared to bacterial sepsis, including differences in the relative contribution of microbial versus host-derived cfDNA and in the temporal evolution of cfDNA signals. Moreover, the lack of standardized cfDNA assays for viral pathogens currently limits direct comparisons across pathogen classes. Future studies incorporating pathogen-stratified cohorts and standardized cfDNA detection approaches will be essential to more comprehensively characterize cfDNA-mediated host–pathogen interactions across different infectious etiologies.

### 4.4 Method-dependent cfDNA correlations with sepsis biomarkers

In line with current concepts of sepsis pathophysiology, our data show that host-derived cfDNA correlates more strongly with inflammatory biomarkers than microbial cfDNA, particularly in early sepsis. Significant associations were found between 90 bp cfDNA fragments and CRP and WBC count, supporting their role as markers of immune activation. These short fragments, typically released during apoptosis and NETosis (19), may serve as sensitive indicators of acute-phase inflammation. The 222 bp cfDNA fragment also correlated significantly with WBC count, potentially reflecting later-stage apoptotic or early necrotic processes and ongoing immune activation. This aligns with previous findings reporting that longer cfDNA fragments increase as inflammation advances (13, 19). A key mediator in this context is endosomal TLR 9, which recognizes both human (DAMP) and microbial cfDNA (PAMP) (**Figure 1**). TLR 9 not only mediates inflammation but also modulates apoptosis. Depending on the context, TLR9 can promote or inhibit programmed cell death, thereby shaping the profile of cfDNA released during sepsis (20–22). In the setting of sepsis, pro-apoptotic TLR9 signaling is likely dominant, contributing to dysregulated immune responses and elevated cfDNA levels. Consistent with this mechanism, the cfDNA integrity index (222/90 ratio) showed a significant negative correlation with CRP, indicating that higher inflammatory activity is associated with a shift toward shorter, highly fragmented cfDNA, suggestive of pronounced NETosis during acute activation. Conversely, higher cfDNA integrity index values, reflecting a relative predominance of longer cfDNA fragments, have been associated with necrotic cell death and more extensive tissue injury, in which DNA is released with less controlled fragmentation. These observations are consistent with recent work demonstrating that cfDNA fragmentation patterns themselves carry important biological information, with shorter fragments reflecting acute inflammatory or apoptotic processes and reduced integrity indices correlating with systemic injury (23). In this context, patients who clinically recover over the course of sepsis would be hypothetically expected to show a temporal shift of the cfDNA integrity index toward higher values, reflecting resolution of acute immune-mediated cell death and a transition away from NETosis-dominated cfDNA release.

In contrast, total human cfDNA reads measured via the iSEP-SEQ assay were significantly associated with LDH and WBC count, indicating a broader signal of tissue injury. As LDH reflects lytic cell death, sequencing-based quantification likely captures cfDNA from diverse forms of cellular damage. The differing correlation patterns between cfDNA measured by qPCR (90 bp assay) and total human reads from sequencing reflect the distinct characteristics of each method. While the qPCR assay targets fragments equal to or longer than 90 bp, providing data on a specific size range, the iSEP-SEQ assays captures a broader, untargeted fragment spectrum from various tissues (11). Thus, cfDNA measured via the 90 bp qPCR assay may predominantly reflect actively circulating cfDNA in acute inflammation, whereas sequencing-derived total read counts may better represent cumulative tissue damage, including endothelial and organ injury.

Although microbial cfDNA reads represented a minor fraction of total cfDNA, they still correlated significantly with CRP, WBC and D-dimer (24). This supports the concept that even low levels of PAMPs can activate innate immunity via PRRs such as TLR9 (5, 16), contributing to cytokine release and leukocyte activation early in sepsis (25). The significant correlation with D-dimer further suggests that microbial cfDNA may be linked to early endothelial dysfunction or sepsis-associated coagulopathy. No relevant correlations were observed between any cfDNA parameters and lactate or platelet counts, indicating that cfDNA release may be more closely tied to inflammation and immune activation than to hypoperfusion or consumptive coagulopathy at the time of sampling.

### 4.5 Comorbidities, vulnerability, and PAMP–DAMP amplification

Comorbidities are highly prevalent among critically ill patients with sepsis and represent an important source of biological variability in cfDNA measurements. Chronic conditions such as cardiovascular disease, chronic kidney disease, diabetes mellitus, and immune-mediated disorders are independently associated with increased sepsis severity and mortality and are common in ICU populations (26), as well as in our cohort (**Table 1**).

These conditions are frequently accompanied by chronic low-grade inflammation, endothelial dysfunction, and immune dysregulation, a phenomenon often described as inflammaging, which may alter baseline innate immune responsiveness and predispose patients to exaggerated inflammatory reactions upon infection (27).

Importantly, despite the potential confounding effect of comorbidities, we observed a strong positive correlation between microbial cfDNA—reflecting bacterial burden—and host-derived cfDNA. This finding suggests that host cfDNA release scales with pathogen-derived signals even in a heterogeneous cohort with varying comorbidity burden. This raises the question of whether comorbidities do not merely confound cfDNA measurements but instead reflect an increased biological vulnerability to microbial insults. In such a scenario, a given pathogen load (PAMPs) may elicit a disproportionately strong host response, resulting in amplified tissue injury, NETosis, and subsequent DAMP release.

From a mechanistic perspective, pre-existing endothelial damage, impaired immune regulation, or reduced organ reserve associated with chronic disease may lower the threshold for PAMP-induced immune activation and facilitate the transition toward DAMP-driven amplification of inflammation. For example, chronic kidney disease has been shown to be associated with persistent activation of innate immune cells, including monocytes and neutrophils, as well as impaired immune regulation (28), which may enhance susceptibility to infection-induced immune dysregulation. Similarly, chronic disease–related immune dysfunction has been linked to impaired pathogen clearance and prolonged inflammatory signaling (29).

Taken together, these observations support a model in which comorbidities may modulate host–pathogen interactions by increasing vulnerability to PAMP-driven immune activation and accelerating the subsequent DAMP-mediated amplification phase. However, due to the limited sample size, stratified analyses by individual comorbidities were not feasible, and this hypothesis requires confirmation in larger, phenotypically well-characterized cohorts.

## 5 Limitations

This study has several limitations. First, the sample size of this exploratory study was small (n = 18), which limits statistical power and the generalizability of the findings. In addition, the single-center design may introduce selection bias, as patient characteristics, disease severity, and treatment strategies may not be representative of broader sepsis populations. The study is therefore not powered to assess associations between cfDNA profiles and clinical outcomes or to conduct meaningful subgroup analyses and the reported correlations should be considered preliminary and hypothesis-generating.

Second, cfDNA was analyzed at a single time point during early sepsis. Given the dynamic nature of the condition, this cross-sectional design does not allow assessment of temporal changes in cfDNA profiles or their relationship to treatment response or disease progression. Longitudinal sampling will be essential in future studies to evaluate the kinetics of cfDNA during sepsis, in particular, to assess how the temporal dynamics of microbial cfDNA relate to human cfDNA, and to evaluate how antimicrobial therapy, organ support, and disease evolution influence cfDNA abundance, composition, and integrity over time.

Third, the observational design precludes causal inference and is subject to confounders by treatment-related factors such as empirical antimicrobial therapy, vasopressor use, mechanical ventilation, and renal replacement therapy, all of which may influence cfDNA release or clearance. Pre-analytical and biological confounders, including impaired renal or hepatic function, systemic inflammation, and variable cfDNA clearance kinetics, may further affect measured cfDNA levels. Further, pre-ICU empirical antimicrobial therapy cannot be excluded and it remains unclear how early treatment affects the initial microbial cfDNA abundance; antibiotic-induced bacterial lysis may either increase or decrease detectable microbial cfDNA abundance. This uncertainty requires further investigation.

Fourth, cfDNA analyses were not stratified by pathogen type, and the cohort predominantly included patients with bacterial sepsis. This limits conclusions regarding pathogen-specific cfDNA dynamics, particularly for viral infections, for which standardized cfDNA detection assays are currently lacking. Further, complementary assays such as 16S qPCR, which could provide additional validation of bacterial cfDNA signals, were not performed.

Finally, due to the small sample size and equal sex distribution, potential effects of comorbidities, age, or sex on cfDNA profiles could not be systematically assessed. Future studies should include larger cohorts and longitudinal sampling to validate and extend these findings.

## 6 Conclusion

In summary, this study demonstrates that cfDNA profiling in early sepsis provides valuable insights into both pathogen detection and host response. The strong concordance between qPCR and iSEP-SEQ confirms the reliability of both methods for quantifying human cfDNA, while the added taxonomic resolution of sequencing allows for simultaneous detection of microbial DNA. Despite the low abundance of microbial cfDNA, its association with key inflammatory markers highlights its relevance in immune activation. Most notably, the consistent predominance and biomarker correlations of host-derived cfDNA support its role as a central amplifier of the septic response. These findings reinforce the concept of PAMP-triggered but DAMP-driven inflammation and suggest that human cfDNA may serve not only as a diagnostic marker but also as a potential target for therapeutic modulation in early sepsis.

## Data Availability

The original contributions presented in the study are included in the article/**Supplementary Material**, further inquiries can be directed to the corresponding author/s.
